# Draft genome sequences of seven endophytic bacterial strains isolated from the roots of coastal plants in Taiwan

**DOI:** 10.1128/mra.00964-25

**Published:** 2025-10-13

**Authors:** Hsi-Ching Yen, Fan-Chen Huang, Pei-Ru Chien, Chih-Lin Wu, Liang-Yu Chen, Chuan-Wen Ho, Guo-Zhang M. Song, Wei-Jen Lin, Hsing-Juh Lin, Chieh-Chen Huang, Hau-Hsuan Hwang, Chih-Horng Kuo

**Affiliations:** 1 Institute of Plant and Microbial Biology, Academia Sinica 71559, Taipei, Taiwan; 2 GeneReach Biotechnology Corp, Taichung, Taiwan; 3 Department of Life Sciences, National Chung Hsing University 34916, Taichung, Taiwan; 4 Department of Soil and Water Conservation, National Chung Hsing University 34916, Taichung, Taiwan; 5 Department of Biological Resources, National Chiayi University 34916, Chiayi, Taiwan; 6 Innovation and Development Center of Sustainable Agriculture, National Chung Hsing University 612476, Taichung, Taiwan; 7 Advanced Plant and Food Crop Biotechnology Center, National Chung Hsing University 34916, Taichung, Taiwan; 8 Biotechnology Center, National Chung Hsing University 34916, Taichung, Taiwan; Wellesley College, Wellesley, Massachusetts, USA

**Keywords:** genome, endophyte, Taiwan

## Abstract

Endophytic bacteria may promote host plant growth and have potential applications in sustainable agriculture. To support the development of these bioresources, we report the draft genome assemblies of seven endophytic bacterial strains isolated from coastal plants in Taiwan.

## ANNOUNCEMENT

Endophytic bacteria confer diverse benefits to host plants by promoting growth, nutrient acquisition, stress tolerance, and pathogen resistance through multiple mechanisms (1). In particular, strains isolated from halophytic plants can enhance host tolerance to salinity and drought, offering potential for improving crop resilience under abiotic stress in sustainable agriculture (2–4). To facilitate bioresource development, we report the draft genome assemblies of seven endophytic bacteria isolated from coastal plants in Taiwan, including halophytes and mangroves adapted to saline and drought stresses (Table 1).

**TABLE 1:** Metadata and genome assembly statistics of the strains included in this study

Strain	Species	Collection date	Location	GPS coordinates	Host	SRA accession	Assembly accession	No. of reads	Coverage (fold)	No. of contigs	Contig N50 (bp)	Assembly size (bp)	GC content (%)	No. of annotated genes	Complete- ness (%)	Contamination (%)
NCHU4564	*Paenarthrobacter* sp.	09 November 2021	Taiwan: Changhua county, Fangyuan township	23.928038 N 120.314438 E	*Portulaca pilosa*	SRR34699254	GCA_052156435.1	5,551,648	176.8	60	171,923	4,527,049	63.9	4,219	99.08	0.21
NCHU4578	*Pantoea ananatis*	09 November 2021	Taiwan: Changhua county, Fangyuan township	23.928038 N 120.314438 E	*Tetragonia tetragonoides*	SRR34699255	GCA_052156415.1	6,114,650	185.5	30	332,022	4,821,288	53.4	4,552	94.57	1.73
NCHU4618	*Pantoea ananatis*	09 November 2021	Taiwan: Changhua county, Fangyuan township	23.928038 N 120.314438 E	*Sesuvium portulacastrum*	SRR34699256	GCA_052156455.1	5,831,752	176.4	33	332,022	4,821,709	53.4	4,553	94.57	1.92
NCHU4620	*Pseudomonas oryzihabitans*	09 November 2021	Taiwan: Changhua county, Fangyuan township	23.928038 N 120.314438 E	*Ipomoea pes-caprae* subsp. *brasiliensis*	SRR34699317	GCA_052156475.1	7,331,544	217.4	27	338,634	4,863,522	66.1	4,538	98.24	0.51
NCHU5208	*Pseudomonas* sp.	04 June 2022	Taiwan: Tainan city, Beimen district	23.294624 N 120.111811 E	*Suaeda maritima*	SRR34699318	GCA_052156515.1	7,388,284	186.8	100	116,682	5,664,563	63.1	5,202	99.95	1.41
NCHU5216	*Pseudomonas* sp.	04 June 2022	Taiwan: Tainan city, Beimen district	23.294624 N 120.111811 E	*Suaeda maritima*	SRR34699353	GCA_052156495.1	6,677,448	169.5	91	112,816	5,666,648	63.1	5,201	99.95	1.41
NCHU5232	*Pseudomonas* sp.	04 June 2022	Taiwan: Tainan city, Beimen district	23.294624 N 120.111811 E	*Avicennia marina*	SRR34699354	GCA_052156555.1	6,494,230	167.2	94	115,768	5,666,451	63.1	5,198	99.95	1.41

Unless stated otherwise, methods followed the cited references. Kits were used according to the manufacturers’ instructions, and bioinformatics tools were used with default settings. The strains were isolated as described previously (5). Briefly, roots were dug out with a clean shovel, soil particles were removed by shaking and rinsing in sterile water, and ∼1 g fresh tissue per plant was surface-sterilized by immersion in 1.2% NaOCl for 20–30 min followed by three rinses in sterile distilled water. The tissues were homogenized in distilled water, serially diluted, spread onto Luria Broth (LB) agar plates, and incubated at 30°C for 48 h. Single colonies were re-streaked on fresh agar plates at least three times to ensure strain purity. For DNA extraction, a single colony of each strain was cultured in 1 mL of liquid LB medium at 30°C for 24 h. DNA was first extracted using the Wizard Genomic DNA Purification Kit (Promega) and further processed using the DNeasy Blood and Tissue Kit (Qiagen) to improve purity.

For shotgun sequencing, DNA library preparation was performed using the SATLite DNA Prep Kit with the SATLite 2.0 Library Prep Automation Device (GeneReach Biotechnology) and sequenced on a NovaSeq X Plus platform using the 10B Reagent Kit (Illumina) to obtain paired-end 150 bp reads. Raw reads were trimmed using fastp v1.0.1 (6), then assembled using SPAdes v3.15.0 (7) with the “--isolate” option (k = 21, 33, 55, and 77). Contigs longer than 500 bp were retained. Trimmed reads were mapped back to the contigs using BWA v0.7.17-r1188 (8), and coverage was assessed using SAMtools v1.9 (9). For species identification, assemblies were compared to reference genomes in the NCBI RefSeq database (10) using FastANI v1.33 (11). Assemblies showing >95% average nucleotide identity to a type strain were assigned to the species level; others were identified to the genus level. Assemblies were submitted to GenBank and annotated using the NCBI Prokaryotic Genome Annotation Pipeline v6.10 (12). Completeness and contamination were estimated using CheckM v1.2.4 (13).

Table 1 lists the metadata (collection date, location, host plant, and species assignment), assembly and annotation statistics, and NCBI accession numbers for each strain. These include one *Paenarthrobacter* sp. strain, two *Pantoea ananatis* strains, and four *Pseudomonas* sp. strains. Contig counts ranged from 27 to 100, N50 ranged from 113 to 339 kb, assembly sizes ranged from 4.53 to 5.67 Mb, GC content ranged from 53.4 to 66.1%, number of annotated genes ranged from 4,219 to 5,202, and all assemblies showed >94.5% completeness and <2% contamination.

## Data Availability

The raw sequencing reads and draft genome assemblies have been deposited in NCBI GenBank and are publicly available; accession numbers are listed in Table 1.
